# Assessment of Reason for Exam Imaging Reporting and Data System (RI-RADS) in inpatient diagnostic imaging referrals

**DOI:** 10.1186/s13244-024-01846-x

**Published:** 2024-11-08

**Authors:** Marco Parillo, Federica Vaccarino, Daniele Vertulli, Gloria Perillo, Edoardo Montanari, Carlo Augusto Mallio, Carlo Cosimo Quattrocchi

**Affiliations:** 1 Radiology, Multizonal Unit of Rovereto and Arco, APSS Provincia Autonoma Di Trento, Trento, Italy; 2 Fondazione Policlinico Universitario Campus Bio-Medico, Roma, Italy; 3 Research Unit of Diagnostic Imaging and Interventional Radiology, Department of Medicine and Surgery, Università Campus Bio-Medico di Roma, Roma, Italy; 4 Centre for Medical Sciences—CISMed, University of Trento, Trento, Italy

**Keywords:** Practice guidelines, Referral and consultation, Reliability and validity, Diagnostic imaging, Radiology

## Abstract

**Objectives:**

To test the Reason for Exam Imaging Reporting and Data System (RI-RADS) in assessing the quality of radiology requests in an Italian cohort of inpatients; to evaluate the interobserver reliability of RI-RADS.

**Methods:**

A single-center quality care study was designed to retrospectively identify consecutive radiology request forms for computed tomography, magnetic resonance imaging, and conventional radiography examinations. One radiologist scored the requests using the RI-RADS. The association between RI-RADS and clinical request variables (urgent request, on-call requests, indication for imaging, requesting specialty, imaging modality, and body region) was evaluated. We calculated interobserver agreement between four readers in a subset of 450 requests.

**Results:**

We included 762 imaging requests. RI-RADS grades A (adequate request), B (barely adequate request), C (considerably limited request), D (deficient request), and X were assigned to 8 (1%), 49 (7%), 237 (31%), 404 (53%), and 64 (8%) of cases, respectively. In the multivariate analysis, the indication for imaging, body region, and requesting specialty significantly influenced the RI-RADS. Indications for imaging with a high risk of poor RI-RADS grade were routine preoperative imaging and device check requests. The upper extremity was the body region with the highest risk of poor RI-RADS grade. Requesting specialties with a high risk of poor RI-RADS grade were cardiovascular surgery, intensive care medicine, and orthopedics. The analysis of the interobserver agreement revealed substantial agreement for the RI-RADS grade.

**Conclusion:**

The majority of radiology exam requests were inadequate according to RI-RADS, especially those for routine imaging. RI-RADS demonstrated substantial reliability, suggesting that it can be satisfactorily employed in clinical settings.

**Critical relevant statement:**

The implementation of RI-RADS can provide a framework for standardizing radiology requests, thereby enabling quality assurance and promoting a culture of quality improvement.

**Key Points:**

- RI-RADS aims to grade the completeness of radiology requests.
- Over half of the imaging requests were RI-RADS D grade; RI-RADS demonstrated substantial reliability.
- Most radiology requests were inadequate and RI-RADS could classify them in clinical practice.

**Graphical Abstract:**

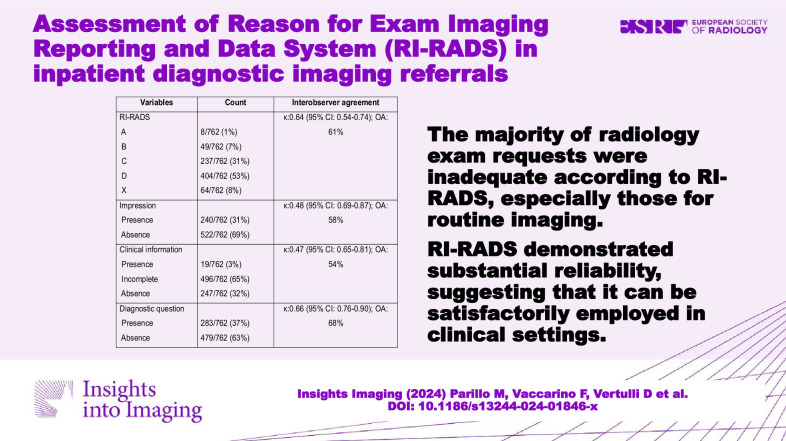

## Introduction

Structured requests for diagnostic imaging referrals are crucial for smooth communication within healthcare. These requests, in fact, streamline the process of referring patients for imaging tests; however, their importance can be overlooked. In some cases, missing clinical details or unrealistic expectations about radiology’s capabilities can lead to miscommunication between doctors requesting the examinations and radiologists interpreting them [1]. Furthermore, to ensure proper justification for imaging tests and minimize patient radiation exposure, radiologists rely on comprehensive information in radiology request forms [2]. Moreover, omitting crucial clinical details on radiology request forms can be a significant contributor to diagnostic errors with potentially serious consequences, including threats to patient safety and malpractice claims [3]. The additional burden of routinely searching medical records or contacting physicians for missing information can significantly hinder workflow efficiency within radiology departments.

A recent development is the Reason for Exam Imaging Reporting and Data System (RI-RADS) which aims to standardize the clinical information included in radiology request forms. This allows radiologists to effectively assess the adequacy of the provided details and transmit their evaluation within the radiology report [4, 5]. Incorporating a system of constructive feedback for suboptimal requests alongside recognition for well-crafted requests could influence physicians towards improved practices, elevating the overall quality of submitted imaging requests. The RI-RADS relies solely on the details within the request form to categorize its completeness. It utilizes a five-point scale to assess the quality of an imaging request, focusing on three key elements: “impression”, “clinical information”, and “diagnostic question” [5].

While prior research suggests a high rate of inadequate radiology request forms (up to 75%) [6], the RI-RADS system’s effectiveness in assessing request quality remains under investigation in different healthcare settings. This study aims to address this gap by evaluating RI-RADS for inpatient imaging referrals in a single Italian healthcare institution. Furthermore, we will investigate interrater agreement with the aim to add evidence and validate this scoring system in the real world [7].

## Methods

### Study design

The study was conducted in accordance with the Declaration of Helsinki (as revised in 2013). The Institutional Review Board deemed this study to be a quality care control investigation, without patient-identifiable information, and therefore exempted it from requiring formal approval or informed consent.

A single-center quality care study was designed with the aim of retrospectively identifying consecutive radiology request forms for computed tomography (CT), magnetic resonance imaging (MRI), and conventional radiography (CR) of inpatients from March 1, 2023, to May 4, 2023. The purpose was to collect a comparable proportion of diagnostic imaging referrals for each of the three imaging modalities (roughly 33% of the overall requests), given the lower volume of MRI requests compared to CR or CT. All imaging referrals originated from a 357-bed secondary care university hospital affiliated with the Italian National Health Service. We focused on inpatients, as their requests were directly integrated into the electronic health record system by the referring physicians.

We extracted the subsequent variables from the institutional radiological information system (Carestream Health, Rochester, New York, United States of America): urgent request (yes or no), on-call request (yes or no), indication for imaging (abdominal disease, central nervous system disease, device check, heart disease, infection, lung disease, musculoskeletal disease, oncology, routine postoperative, routine preoperative, postoperative complication, stroke, or unspecified), requesting specialty (cardiology, cardiovascular surgery, emergency medicine, gastroenterology, general surgery, geriatrics, intensive care medicine, internal medicine, neurology, oncology, orthopedics, thoracic surgery, or miscellaneous), imaging modality (CR, CT, or MRI), body region (abdomen, central nervous system, chest, chest and abdomen, heart, lower extremity, pelvis, spine, upper extremity, whole-body, or other multiple body regions), and contrast medium injection for CT or MRI scans (yes or no).

### RI-RADS evaluation

One radiologist assessed all imaging requests included and assigned a score using the RI-RADS. Only the in-hospital requisition forms, which were digital and unstructured, were used for this assessment. No information from other sources, like electronic medical records, was considered. Three key categories determined the RI-RADS grade: (1) impression, (2) clinical information, and (3) diagnostic questions. The scoring system itself has five categories (A, B, C, D, or X), with A indicating the complete request and X signifying the most deficient one (refer to Table 1 for details) [5]. To assess interobserver agreement, three radiology residents (with 3 years of experience) independently assigned RI-RADS grade to the first 150 requests for CR, CT, and MRI scans (each resident reviewed a total of 450 requests).

**Table 1:** Key categories and examples in RI-RADS evaluation

Key categories			RI-RADS grading*
Impression	Clinical information	Diagnostic question	
Working or differential diagnosis	Signs and symptoms, chronicity of current episode, location of signs and symptoms, pertinent past medical/surgical history, pertinent laboratory findings, and previous imaging reports when available	Confirmation/exclusion of diagnosis, grading/staging, pre-operative planning, follow-up of progress, or response to treatment	• A (adequate request; e.g., urgent contrast-enhanced abdominal CT scan in a patient on the 26th postoperative day following right hemicolectomy for perforation of unknown etiology with worsening acute abdominal pain. Quantification of free air and fluid and exclusion of bowel ischemia) • B (barely adequate request; e.g., contrast-enhanced abdominal CT for suspected bowel ischemia in a patient on Xth postoperative day following subtotal colectomy, with lactate level of 98 mg/dL and bloody drainage) • C (considerably limited request; e.g., chest X-ray for desaturation in a patient with pleural drainage) • D (deficient request; e.g., chest X-ray for tube check) • X (e.g., chest X-ray)

^*^ RI-RADS A indicates the presence of all three key categories, RI-RADS B signifies the presence of all three categories with some clinical details missing, RI-RADS C denotes the availability of only two key categories, and RI-RADS D signifies the presence of just one. RI-RADS X represents a scenario where no key category of information is available

*CT* computed tomography

### Statistical analysis

To assess how consistently the four readers assigned RI-RADS scores, we calculated interobserver agreement and Fleiss’ kappa (κ) in a subset of 450 requests. This analysis considered both the overall score (A, B, C, D, or X) and each of the three key components used to determine the score (impression, clinical information, and diagnostic question). The κ statistic was interpreted using a six-point scale: less than 0 indicates no agreement, 0.01–0.20 suggests slight agreement, 0.21–0.40 represents a fair agreement, 0.41–0.60 indicates moderate agreement, 0.61–0.80 signifies substantial agreement, and 0.81–0.99 suggests near-perfect agreement [8, 9].

The association between RI-RADS grades and each of the clinical request variables (urgent request, on-call requests, indication for imaging, requesting specialty, imaging modality, and body region) was tested using the Chi-squared test for independence. After conducting the tests, the Bonferroni correction was applied to adjust for multiple comparisons, setting the significance threshold at 0.05 divided by the number of tests performed (six in this case, corresponding to a corrected alpha of 0.01). Variables that remained significant after adjustment for multiple testing were tested for collinearity, considering a Cramér’s *V* coefficient > 0.6 as positive for collinearity. Variables identified as having no collinearity were then included in a multivariate model. In the multivariate ordinal regression, the category with the highest prevalence was chosen as the reference group for interpreting the odds ratios (ORs). We further analyzed the CT and MRI subgroups using Chi-squared tests and Cramér’s *V* coefficients to assess the relationship between contrast medium injection and RI-RADS grade. Statistical significance was set at a *p*-value threshold of 0.05.

## Results

### Study population

A total of 762 imaging requests were included in the study (257 CR, 246 CT, and 259 MRI). Most of the requests were non-urgent (88%) and not on-call (93%). The most prevalent imaging indications were routine postoperative (18%), oncology (17%), and stroke (16%). The most prevalent requesting specialties were neurology (15%), internal medicine (13%), and orthopedics (12%). The most prevalent body regions examined were chest (31%), central nervous system (27%), and abdomen (15%). Slightly more than half of the CT and MRI scans were requested with contrast medium administration (53%). Table 2 summarizes the data included in the study.

**Table 2:** Distribution of data included in the study

Variables	Count
Total imaging requests	762
Urgent request	
Yes	94/762 (12%)
No	668/762 (88%)
On-call request	
Yes	50/762 (7%)
No	712/762 (93%)
Indication for imaging	
Abdominal disease	29/762 (4%)
Central nervous system disease (other than stroke)	26/762 (3%)
Device check	52/762 (7%)
Heart disease	10/762 (1%)
Infection	58/762 (8%)
Lung disease	35/762 (4%)
Musculoskeletal disease	19/762 (2%)
Oncology	127/762 (17%)
Routine postoperative	135/762 (18%)
Routine preoperative	35/762 (5%)
Postoperative complication	46/762 (6%)
Stroke	126/762 (16%)
Unspecified	64/762 (8%)
Requesting specialty	
Cardiology	39/762 (5%)
Cardiovascular surgery	87/762 (11%)
Emergency medicine	21/762 (3%)
Gastroenterology	24/762 (4%)
General surgery	54/762 (7%)
Geriatrics	33/762 (4%)
Intensive care medicine	63/762 (8%)
Internal medicine	98/762 (13%)
Neurology	117/762 (15%)
Oncology	63/762 (8%)
Orthopedics	91/762 (12%)
Thoracic surgery	23/762 (4%)
Miscellaneous	49/762 (6%)
Imaging modality	
CR	257/762 (34%)
CT	246/762 (32%)
MRI	259/762 (34%)
Body region	
Abdomen	116/762 (15%)
Central nervous system	202/762 (27%)
Chest	235/762 (31%)
Chest and abdomen	23/762 (3%)
Heart	19/762 (3%)
Lower extremity	40/762 (5%)
Pelvis	54/762 (7%)
Spine	18/762 (2%)
Upper extremity	10/762 (1%)
Whole-body	31/762 (4%)
Other multiple-body regions	14/762 (2%)
Contrast medium (CT or MRI)	
Yes	269/505 (53%)
No	236/505 (47%)

*CR* conventional radiography, *MRI* magnetic resonance imaging, *CT* computed tomography

### RI-RADS grades

Most requests received a RI-RADS grade of D (53%). Furthermore, analysis of the key scoring categories revealed missing impressions and diagnostic questions in most cases (69% and 63%, respectively), while clinical information was mostly incomplete (65%). Subgroup distribution of RI-RADS grades by imaging technique showed a high rate of RI-RADS D in CR (215/257, 84%) and a similar rate of RI-RADS C and D in CT (48%, 118/246 and 33%, 80/246, respectively) and MRI (42%, 109/259 and 42%, 109/259, respectively).

The analysis of the interobserver agreement revealed substantial agreement for RI-RADS grade and diagnostic question category, and moderate agreement for impression and clinical information categories. All the readers agreed on the RI-RADS assignment in 273/450 (61%) requests, three readers agreed in 159/450 (35%) requests, and two readers agreed in 18/450 (4%) requests. In no case, did we find a complete disagreement among the readers. Subgroup agreement analysis by imaging technique showed higher agreement in scoring CR (κ: 0.73; 95% CI: 0.59–0.85) than CT (κ: 0.55; 95% CI: 0.47–0.62) or MRI (κ: 0.54; 95% CI: 0.47–0.62) requests. Table 3 summarizes the distribution of RI-RADS and key categories assigned in the study, along with the values of the interobserver agreement.

**Table 3:** RI-RADS and key categories assigned in the study, along with the values of the interobserver agreement

Variables	Count	Interobserver agreement^*^
RI-RADS		κ: 0.64 (95% CI: 0.54–0.74); OA: 61%
A	8/762 (1%)	
B	49/762 (7%)	
C	237/762 (31%)	
D	404/762 (53%)	
X	64/762 (8%)	
Impression		κ: 0.48 (95% CI: 0.69–0.87); OA: 58%
Presence	240/762 (31%)	
Absence	522/762 (69%)	
Clinical information		κ: 0.47 (95% CI: 0.65–0.81); OA: 54%
Presence	19/762 (3%)	
Incomplete	496/762 (65%)	
Absence	247/762 (32%)	
Diagnostic question		κ: 0.66 (95% CI: 0.76–0.90); OA: 68%
Presence	283/762 (37%)	
Absence	479/762 (63%)	

*κ* Fleiss’ kappa, *CI* confidence interval, *OA* overall agreement

^*^ Performed on a subset of 450 requests by four readers

### RI-RADS grades and variables associations

The Chi-squared tests before the Bonferroni adjustment suggested significant associations between RI-RADS grades and all tested variables except for on-call status. However, after applying the Bonferroni correction, only the associations with an indication for imaging, requesting specialty, imaging modality, and body region remained statistically significant (*p* < 0.01). Among these variables, there was collinearity between indication for imaging and imaging modality (Cramér’s *V* = 0.69) and between imaging modality and body region (Cramér’s *V* = 0.65). Thus, imaging modality was removed from further analysis to reduce the risk of multicollinearity impacting the results. In the multivariate analysis, the indication for imaging, body region, and requesting specialty significantly influenced the RI-RADS grade (*p* < 0.01). Specifically, indications for imaging with high risk of poor RI-RADS grade (excluding requests without specific clinical indication) were routine preoperative imaging (OR: 2.06, *p* < 0.01) and device check (OR: 1.63, *p* < 0.01) requests, while low risk was found for heart disease (OR: 0.24, *p* < 0.01) and postoperative complication (OR: 0.27, *p* < 0.01) requests. The upper extremity was the body region with the highest risk of poor RI-RADS grade, although it was not statistically significant (OR: 2.38, *p* = 0.07). Requesting specialties with a high risk of poor RI-RADS grade were cardiovascular surgery (OR: 2.60, *p* < 0.01), intensive care medicine (OR: 2.37, *p* < 0.01), and orthopedics (OR: 2.40, *p* < 0.01), while low risk was found for emergency medicine (OR: 0.26, *p* < 0.01) (Table 4). Subgroup analysis of CT and MRI scans indicated a statistically significant association between the use of contrast medium and RI-RADS grading (*p* < 0.05). However, Cramér’s *V* coefficient (0.17) suggested that the correlation between these two variables was weak.

**Table 4:** Multivariate ordinal regression analyses show the odds ratio of receiving a low RI-RADS score in relation to indication for imaging, body region, and requesting specialty

Variables	Category	OR	95% CI	*ρ*-value
Indication for imaging	Abdominal disease	0.68	0.38–1.24	0.21
	Central nervous system disease (other than stroke)	0.52	0.28–1.00	**0.04**
	Device check	1.63	1.11–2.41	**0.01**
	Heart disease	0.24	0.10–0.59	**0.00**
	Infection	0.32	0.19–0.52	**0.00**
	Lung disease	0.43	0.26–0.74	**0.00**
	Musculoskeletal disease	0.60	0.32–1.11	0.10
	Oncology	0.41	0.25–0.67	**0.00**
	Routine postoperative	Reference	–	–
	Routine preoperative	2.06	1.29–3.31	**0.00**
	Postoperative complication	0.27	0.16–0.45	**0.00**
	Stroke	0.81	0.47–1.39	0.44
	Unspecified	9.71	6.53–14.44	**0.00**
Body region	Abdomen	0.68	0.48–0.96	**0.03**
	Central nervous system	0.75	0.49–1.15	0.19
	Chest	Reference	–	–
	Chest and abdomen	0.98	0.61–1.58	0.92
	Heart	0.44	0.22–0.90	**0.02**
	Lower extremity	0.81	0.49–1.33	0.40
	Pelvis	1.18	0.70–1.97	0.53
	Spine	0.69	0.38–1.24	0.21
	Upper extremity	2.03	0.95–4.37	0.07
	Whole-body	1.19	0.72–1.96	0.49
	Other multiple-body regions	0.73	0.41–1.32	0.30
Requesting specialty	Cardiology	0.44	0.28–0.70	**0.00**
	Cardiovascular surgery	2.60	1.75–3.86	**0.00**
	Emergency medicine	0.26	0.15–0.43	**0.00**
	Gastroenterology	0.83	0.49–1.39	0.47
	General surgery	0.63	0.42–0.94	**0.02**
	Geriatrics	0.58	0.38–0.90	**0.02**
	Intensive care medicine	2.37	1.63–3.44	**0.00**
	Internal medicine	0.57	0.41–0.79	**0.00**
	Neurology	Reference	–	–
	Oncology	0.55	0.38–0.79	**0.00**
	Orthopedics	2.40	1.41–4.11	**0.00**
	Thoracic surgery	1.24	0.72–2.14	0.44
	Miscellaneous	0.45	0.30–0.67	**0.00**

The category with the highest frequency of observations served as the reference point for comparison. Statistically significant associations (*ρ*-value < 0.05) are highlighted in bold

*CI* confidence interval, *OR* odd ratio

## Discussion

Our analysis revealed that over half (53%) of inpatient imaging requests fell into the RI-RADS D grade, indicating significant deficiencies in terms of completeness; a substantial proportion (92%) reached only RI-RADS C, D, or X, indicating overall low quality. In particular, requests for routine imaging examinations were at a higher risk of being incomplete. Furthermore, RI-RADS demonstrated substantial reliability (κ: 0.64), suggesting that it can be satisfactorily employed in clinical settings to classify the completeness of radiology requests.

Our study found that requests for common preoperative imaging (e.g., chest CR or scans for visualizing anatomy) and device checks (e.g., chest CR to check pleural drains or catheters) were more likely to be incomplete. This could be because the purpose of these examinations seems obvious, leading to less effort in crafting a detailed request. However, even for these routine procedures, clear physician input is essential. Including a well-defined clinical question, relevant clinical information, and the physician’s impression of the imaging request can reduce the risk of diagnostic errors [10]. Indeed, without a broader understanding of the patient’s clinical background, radiologists might miss significant incidental findings unrelated to the diagnostic question. Imaging requests for heart disease and with heart as body region tended to be of higher quality. This might be because these exams, such as cardiac CT or MRI, require specific clinical indications and major communication with cardiology departments, which, in our study, demonstrated a lower rate of incomplete requests compared to other specialties. Imaging requests motivated by postoperative complications, or infections were also associated with higher quality. This comes from the fact that these situations often involve critically ill patients, where a timely and accurate diagnosis is crucial for initiating appropriate treatment. The urgency of these cases likely translates into more focused and detailed imaging requests from physicians. Imaging requests for oncological purposes exhibited a low risk of being incomplete, probably in relation to the inherent complexity of managing cancer patients. Accurate staging and re-staging of cancers often require a comprehensive medical history, which likely translates to more thorough imaging requests from physicians. Among the body regions, upper extremity imaging requests had a higher OR of receiving a low RI-RADS grade although not statistically significant. This might be because these requests often involve routine post-operative examinations from the orthopedic department, which in our study showed a higher tendency for submitting incomplete requests. Cardiovascular surgery and intensive care medicine also exhibited a higher likelihood of low RI-RADS grades, because most of their requests were for routine post-operative procedures or device checks (e.g., chest CR after coronary artery bypass graft or for central venous line check). Our findings align with prior research, which has documented a high prevalence of poorly formulated imaging requests [11–15]. A recent survey, for instance, found that over half of radiologists (56.3%) encounter imaging referrals with missing or inaccurate information, making interpretation challenging in a significant portion of cases. Moreover, about 13% of radiologists reported at least one lawsuit that could have potentially been avoided if the imaging request had contained sufficient clinical information [5]. In particular, our findings align with those of Kasalak et al, who reported a high prevalence of inappropriate radiology requests in a Dutch hospital, despite a different distribution across RI-RADS grades (RI-RADS A, B, C, and D were assigned in 23%, 25%, 32%, and 20% of cases, respectively) [6]. Furthermore, Kasalak et al also observed that the risk of incomplete radiology requests increased when exams were ordered for routine procedures. On the other hand, we identified several variables that were differently associated with the OR of a low RI-RADS grade. For instance, the body region “upper extremity” was associated with a lower risk of incomplete radiology requests in the Dutch study, while in our study, it was associated with a higher risk. Intensive care medicine as a requesting specialty was associated with a high risk of low-quality diagnostic imaging referrals in our sample, while it had a lower risk in the study by Kasalak et al [6]. These discrepancies are likely due to various factors, including: (1) different study samples because our study focused only on CR, CT, and MRI for hospitalized patients, while Kasalak’s study primarily involved outpatients and also included ultrasound imaging; (2) varying hospital settings as demonstrated by the presence of transplant indications and a pediatric department in the Dutch study (significantly associated with RI-RADS grading), but not present in our hospital; and (3) dissimilarity in interactions and collaborations between physicians from different departments and radiologists that may vary between institutions (as demonstrated by the low risk of incomplete cardiac imaging requests from the cardiology department in our study). Additional studies in different clinical contexts could further elucidate these and other aspects that may affect the quality of a radiology request.

Our study further investigated the reliability of RI-RADS with four observers, considering not only the final grade but also the three individual key categories. We found substantial RI-RADS reproducibility, similar to that reported by Kasalak et al, although the latter study assessed agreement only between two readers [6]. The analysis of subcategories revealed moderate agreement for “impression” and “clinical information”, highlighting the ongoing uncertainty in determining whether certain information in radiology requests should be classified under one or the other subcategory. Furthermore, subgroup agreement analysis by imaging technique showed higher agreement in scoring CR than CT or MRI, reflecting the varying complexity of imaging modalities. In CR, routine examinations with standardized requests for multiple patients are prevalent, while CT and MRI involve a wider range of request variations. Introducing more RI-RADS examples for each key category or for CT and MRI techniques could increase agreement. Moreover, as healthcare providers become more familiar with RI-RADS criteria, interrater agreement is likely to improve. A key factor potentially driving RI-RADS adoption could come from artificial intelligence. By leveraging the impressive text analysis capabilities of large language models (LLMs) [16], physicians could potentially input their radiology request in the hospital’s electronic ordering system and receive the RI-RADS grade in real-time, enabling them to adjust the completeness of the request accordingly. Recent research in April 2024 assessed ChatGPT 4’s proficiency in categorizing imaging referrals using the RI-RADS framework, resulting in a low reliability (κ: 0.20) [17]. It is noteworthy that the field of LLMs is rapidly advancing, with frequent updates. Moreover, LLMs possess the capacity to learn from the data they process, potentially leading to variations in responses to identical prompts over time. Future studies could explore the reliability of various LLMs in assigning RI-RADS scores to radiology exam requests across diverse clinical contexts.

The association between RI-RADS grades and each of the clinical request variables warrants cautious interpretation due to several limitations, which, however, do not affect the assessment of interreader agreement since it is independent of these variables. Radiology requests might not capture all the information relayed by referring physicians since oral communication channels could also be used to transmit additional details. However, written imaging requests should include all essential information to minimize the potential for errors and miscommunication. The generalizability of the findings is limited by the single-center study design, making the results not fully applicable to institutions with significantly different examination volumes or types. Our focus on hospitalized patients restricts its application to other settings, like outpatients or emergency department patients. Moreover, the inpatients studied represented a diverse population with varying needs and requests, making direct comparisons to other settings challenging. However, including a consecutive series of imaging referrals provided a more authentic reflection of the imaging requests encountered by a radiologist in routine clinical practice. Finally, the possibility of unidentified variables influencing request completeness cannot be excluded.

## Conclusion

The majority of radiology exam requests were inadequate according to RI-RADS, with RI-RADS D (deficient request) being the most prevalent category (53%). In particular, requests for routine imaging examinations were at a higher risk of being incomplete. Furthermore, RI-RADS demonstrated substantial reliability, suggesting that it can be satisfactorily employed in clinical settings to classify the completeness of radiology requests and implement policies for continuous improvement.

## Data Availability

The data that support the findings of this study are available from the corresponding author, upon reasonable request.

## Abbreviations

CR: Conventional radiography
CT: Computed tomography
LLM: Large language model
MRI: Magnetic resonance imaging
OR: Odds ratio
RI-RADS: Reason for Exam Imaging Reporting and Data System
κ: Fleiss’ kappa
